# Patient mortality exceeds implant revision risk with equivalent survival following unicompartmental versus total knee arthroplasty in octogenarians

**DOI:** 10.1007/s00402-026-06466-2

**Published:** 2026-08-06

**Authors:** Matteo Innocenti, Filippo Leggieri, Marta Massenzi, Davide Stimolo, Mustafa Akkaya, Fabrizio Matassi, Roberto Civinini

**Affiliations:** 1 Department of Clinical Orthopaedics, University of Florence, Florence, Italy; 2 Department of Orthopaedics and Traumatology, Yüksek İhtisas Üniversitesi, Ankara, Turkey

**Keywords:** Octogenarians, Unicompartmental knee arthroplasty, Total knee arthroplasty, Mortality, Competing risk, Implant survivorship

## Abstract

**Background:**

While traditional surgical decision-making emphasizes long-term implant survivorship for octogenarians knee reconstruction, this paradigm may be inappropriate when patient mortality risk substantially exceeds revision risk. This study compared mid-term mortality between unicompartmental knee arthroplasty (UKA) and total knee arthroplasty (TKA) in octogenarians, hypothesizing that implant type would not significantly influence patient survival.

**Methods:**

A retrospective cohort study was conducted on 238 consecutive octogenarian patients (age ≥ 80 years) who underwent primary knee arthroplasty at a single tertiary orthopaedic center between January 2018 and December 2020. Patients with revision arthroplasty, fracture, tumor, inflammatory arthropathy, or incomplete data were excluded. The primary outcome was all-cause mortality at minimum 5-year follow-up. Secondary outcomes included age-stratified mortality (80–84, 85–89, ≥ 90 years) and identification of independent mortality predictors. Statistical analyses included independent t-tests, chi-square/Fisher’s exact tests, standardized mean differences, and multivariable logistic regression adjusting for age, follow-up duration, and registry year.

**Results:**

Of 238 patients, 185 (77.7%) underwent TKA and 53 (22.3%) underwent UKA. Overall mortality was 35.3% (84/238): 33.5% in TKA versus 41.5% in UKA (OR = 1.41, 95% CI: 0.75–2.63, *p* = 0.329). Age-stratified mortality showed no significant differences: 80–84 years (31.2% TKA vs. 37.2% UKA, *p* = 0.467), 85–89 years (42.3% vs. 50.0%, *p* = 1.000). Multivariable analysis identified age as the only significant mortality predictor (adjusted OR = 1.16 per year, 95% CI: 1.03–1.32, *p* = 0.013), while implant type was not associated with mortality (adjusted OR = 1.40, 95% CI: 0.72–2.73, *p* = 0.319). Patient mortality exceeded registry-reported revision rates 3–5 fold.

**Conclusions:**

In octogenarians, patient mortality substantially exceeds implant revision risk, with no significant difference between UKA and TKA. Surgeons should prioritize patient-specific clinical indications over theoretical implant durability concerns when selecting procedures in this population.

**Level of evidence:**

III.

## Introduction

The global population is aging at an unprecedented rate, with individuals aged 80 years and older representing the fastest-growing demographic segment worldwide [1–4]. This demographic shift has profound implications for orthopaedic surgery, as octogenarians now constitute an increasing proportion of knee arthroplasty patients, challenging traditional surgical paradigms developed primarily for younger populations [5–8].

Both unicompartmental knee arthroplasty (UKA) and total knee arthroplasty (TKA) are established surgical options for end-stage knee osteoarthritis. While TKA has historically been favoured for its comprehensive joint resurfacing and perceived durability, UKA offers a less invasive alternative with bone preservation, faster rehabilitation, and superior functional outcomes in appropriately selected patients [9–12]. However, a debate regarding optimal implant selection in octogenarians may rise, weighing the superior implant survivorship of TKA against the reduced surgical trauma and accelerated recovery of UKA.

This debate is predicated on a critical assumption that may not hold true for very elderly patients. Traditional survivorship studies emphasize long-term implant durability, typically reporting 10- to 15-year outcomes, with TKA demonstrating 95–98% survival compared to 90–95% for UKA [13–16].

However, these metrics may become increasingly irrelevant when patient life expectancy is limited. In octogenarians, where 5-year mortality approaches 40–60% according to population-based data [17, 18], the likelihood that a patient will outlive their prosthesis diminishes substantially. Despite this reality, existing literature predominantly focuses on implant-related outcomes while overlooking the competing risk of patient mortality.

While previous studies have examined mortality in elderly knee arthroplasty patients [19–21], none directly compared UKA versus TKA mortality specifically in octogenarians. This gap is clinically significant because surgical decision-making in elderly patients should prioritize short-term outcomes, perioperative safety, and functional recovery over theoretical long-term implant durability. If UKA and TKA demonstrate similar mortality profiles in octogenarians, the less invasive nature of UKA—with its associated benefits of reduced blood loss, shorter operative time, and accelerated mobilization—may represent a more rational choice for this vulnerable population.

The aim of this study was to compare 5-year mortality rates between octogenarians undergoing UKA versus TKA, hypothesizing that implant type would not significantly influence patient survival. We further aimed to identify the primary predictors of mortality in this population and to determine whether age-stratified subgroups demonstrated differential mortality patterns between procedures.

We hypothesized that: (1) there would be no significant difference in mortality between UKA and TKA at mid-term follow-up; (2) patient age, rather than implant type, would emerge as the dominant predictor of mortality; and (3) the overall mortality rate would substantially exceed typical implant failure rates reported in the literature, supporting the clinical equipoise for UKA use in carefully selected octogenarians. By shifting focus from implant survivorship to patient survivorship, this study addresses a critical evidence gap and provides data to inform more appropriate surgical decision-making in our aging population.

## Materials and methods

### Design and population

A retrospective cohort study was conducted on a consecutive series of octogenarian patients (age ≥ 80 years) who underwent primary knee arthroplasty at a single tertiary orthopaedic center between January 2018 and December 2020. The study was performed in accordance with the Declaration of Helsinki and approved by the local institutional review board. Given the retrospective nature of the study, the requirement for informed consent was waived. The study was conducted according to the Strengthening the Reporting of Observational Studies in Epidemiology (STROBE) guidelines for cohort studies.

All patients aged 80 years or older at the time of surgery who underwent primary elective unicompartmental knee arthroplasty (UKA) or total knee arthroplasty (TKA) - ICD9 81.45 - were assessed for eligibility. Patients were excluded if they had: (1) revision arthroplasty, (2) arthroplasty for fracture, tumor, or inflammatory arthropathy, or (3) incomplete demographic or follow-up data. All procedures were performed by high-volume arthroplasty surgeons at a high-volume institution. Implant selection (UKA versus TKA) was based on patient-specific factors including osteoarthritis pattern, ligamentous stability, deformity severity, and surgeon preference, following established clinical indications for unicompartmental versus total knee replacement.

### Data collection and study endpoints

Baseline demographics were extracted from the institutional registry, including age at surgery (years), surgical side (left/right), date of surgery, and registry year (2018–2020). The primary exposure variable was implant type, classified as UKA or TKA, extracted from operative records. Age was analysed both as a continuous variable and categorized into subgroups: 80–84 years, 85–89 years, and ≥ 90 years. Mortality status was ascertained for all patients through 31 st December 2025, providing a minimum follow-up of 5 years for all included patients. The date of death was not available from registry data; therefore, patients were classified as deceased (yes/no) at the end of follow-up.

### Outcome measures

The primary outcome was all-cause mortality at the end of follow-up. Secondary outcomes included mortality stratified by age category (80–84, 85–89, ≥ 90 years), and identification of independent predictors of mortality in this population.

### Statistical analysis

Descriptive statistics were calculated for all variables. Continuous variables were reported as means with standard deviations (SD) and compared between UKA and TKA groups using independent t-tests. Categorical variables were presented as frequencies and percentages and compared using chi-square tests or Fisher’s exact tests when cell counts were < 5. Standardized mean differences (SMD) were calculated to assess baseline imbalance between groups, with SMD > 0.1 indicating meaningful differences. Mortality rates were calculated as proportions with 95% confidence intervals (CI). Crude associations between implant type and mortality were assessed using chi-square tests and odds ratios (OR) with 95% CI. Multivariable logistic regression models were constructed to identify independent predictors of mortality, adjusting for potential confounders. The following covariates were included: implant type (UKA vs. TKA), age at surgery (continuous), follow-up duration (continuous), and registry year (2018–2020). Results were reported as adjusted ORs with 95% CI and P-values. Subgroup analyses were performed stratified by age category (80–84, 85–89, ≥ 90 years) to assess whether the association between implant type and mortality differed across age strata. Within each age stratum, mortality rates were compared using Fisher’s exact tests. All statistical analyses were performed using SPSS statistical software version 21.1 (IBM Corporation, NY, USA). Statistical significance was set at *P* < 0.05 for all analyses.

## Results

### Study population and baseline characteristics

A total of 238 octogenarian patients who underwent primary knee arthroplasty between 2018 and 2020 met the inclusion criteria and were included in the analysis. Of these, 185 (77.7%) underwent TKA and 53 (22.3%) underwent UKA. Baseline patient characteristics stratified by implant type are presented in Table 1. The TKA group was slightly older than the UKA group (mean age 82.9 vs. 82.2 years, *p* = 0.047), though the absolute difference was small. The groups were well-balanced with respect to age at surgery and follow-up duration, with no significant differences detected. However, a trend toward difference was observed in the distribution of age categories (*p* = 0.048), while registry year showed significant differences (*p* = 0.017).

**Table 1:** Baseline characteristics of study population stratified by implant type

Variable	TKA	UKA	*p*_value	SMD
Age at surgery (years), mean (SD)	82.9 (2.3)	82.2 (2.5)	0.047	0.321
Follow-up duration (years), mean (SD)	6.2 (0.9)	6.3 (0.7)	0.386	0.128
Age Category, n (%)			0.05	0.322
80–84 years	157 (84.9)	43 (81.1)		
85–89 years	26 (14.1)	6 (11.3)		
≥90 years	2 (1.1)	4 (7.5)		
Registry Year, n (%)			0.017	0.451
2018	50 (27.0)	10 (18.9)		
2019	71 (38.4)	32 (60.4)		
2020	64 (34.6)	11 (20.8)		
Surgical Side, n (%)			0.886	0.047
Left	76 (41.1)	23 (43.4)		
Right	109 (58.9)	30 (56.6)		

*SMD S*tandardized Mean Difference*, SD *Standard Deviation

### Overall mortality

At the end of follow-up, 84 patients (35.3%) had died. Mortality rates stratified by implant type are presented in Table 2. The crude mortality rate was 33.5% in the TKA group and 41.5% in the UKA group, with no statistically significant difference between groups (*p* = 0.329, Fisher’s exact test).

**Table 2:** Overall mortality rates by implant type

Implant_Type	*N*	Deaths	Alive	Crude_OR	*p*_value
TKA	185	62 (33.5)	123 (66.5)	Reference	-
UKA	53	22 (41.5)	31 (58.5)	1.41 (0.75–2.63)	0.329
Total	238	84 (35.3)	154 (64.7)		

*OR *Odds Ratio*, CI *Confidence Interval

### Mortality stratified by age category

Mortality rates increased substantially with advancing age, as shown in Table 3. Among patients aged 80–84 years, mortality was similar between UKA and TKA groups, with no significant difference (37.2% vs. 31.2%, *p* = 0.467). In the 85–89 year age group, the crude mortality rate was numerically higher in the UKA group, though this difference was not statistically significant (50.0% vs. 42.3%, *p* = 1.000). Sample sizes were insufficient for statistical comparison in the ≥ 90 year subgroup.

**Table 3:** Mortality rates stratified by age category and implant type

Age_Category	Implant	*N*	Deaths	Crude_OR	*p*_value
80–84 years	TKA	157	49 (31.2)	Reference	
	UKA	43	16 (37.2)	1.31 (0.65–2.64)	0.467
85–89 years	TKA	26	11 (42.3)	Reference	
	UKA	6	3 (50.0)	1.36 (0.23–8.08)	1
≥ 90 years	TKA	2	2 (100.0)	-	
	UKA	4	3 (75.0)	-	-

*OR = *UKA compared to TKA as reference

### Predictors of mortality

Univariate and multivariate logistic regression analyses identified age at surgery identified age at surgery as the primary significant predictor of mortality (Table 4). Age remained the strongest independent predictor of mortality, with each additional year of age associated with a 16% increase in mortality risk (adjusted OR = 1.16, 95% CI: 1.03–1.32, *p* = 0.013) after adjusting for other covariates. Follow-up duration was significantly associated with mortality (OR = 1.57 per year, 95% CI: 1.13–2.21, *p* = 0.008; univariate analysis), though this association did not remain statistically significant in the multivariate model (adjusted OR = 1.53, 95% CI: 0.64–3.66, *p* = 0.339). implant type was not independently associated with mortality in either univariate (OR = 1.41, 95% CI: 0.75–2.63, *p* = 0.284) or multivariable analysis after adjusting for age, follow-up duration, and registry year (adjusted OR = 1.40, 95% CI: 0.72–2.73, *p* = 0.319), indicating no significant difference in mortality risk between UKA and TKA procedures.

**Table 4:** Univariate logistic regression analysis for predictors of mortality

Variable	Uni_OR	Uni_CI	Uni_p	Multi_OR	Multi_CI	Multi_p
Implant Type (UKA vs. TKA)	1.41	0.75–2.63	0.284	1.4	0.72–2.73	0.319
Age at Surgery (per year)	1.14	1.02–1.28	0.022	1.16	1.03–1.32	0.013
Follow-up Duration (per year)	1.57	1.13–2.21	0.008	1.53	0.64–3.66	0.339
Registry Year (2019 vs. 2018)	1	0.53–1.92	0.992	1.51	0.50–4.60	0.463
Registry Year (2020 vs. 2018)	0.38	0.18–0.80	0.012	0.88	0.13–5.81	0.896

*OR *Odds Ratio, *CI* Confidence Interval, Model AIC = 303.7; C-statistic = 0.65

In age-stratified analyses with adjustment for follow-up duration, no significant association was observed between implant type and mortality in any age subgroup. Among patients aged 80–84 years, the adjusted OR for UKA versus TKA was 1.30 (95% CI: 0.63–2.64, *p* = 0.467). In the 85–89 year subgroup, the adjusted OR was 1.15 (95% CI: 0.17–7.77, *p* = 0.883), though this estimate was imprecise due to small sample size. The ≥ 90 year subgroup had insufficient sample size for adjusted analysis.

## Discussion

We found no significant difference in mid-term mortality between UKA and TKA procedures. Age emerged as the dominant predictor of mortality, with each additional year conferring a 16% increase in mortality risk. These findings—representing the first direct mortality comparison between UKA and TKA exclusively in octogenarians—supported the clinical equipoise between the procedures with respect to patient survival and explicitly contextualized implant selection within the competing risk framework of patient survival versus implant durability.

The overall mortality rate of 35.3% was consistent with population-based life expectancy data for octogenarians and aligned with prior studies in elderly surgical populations. Our age-stratified mortality rates demonstrated the steep mortality gradient in this population, underscoring the competing risk that patient death poses relative to implant failure.

Our findings confirmed and extended previous comparative mortality studies between UKA and TKA that were based on the broader arthroplasty population. Hunt et al. [20] found significantly lower 90-day mortality following UKA compared to TKA. Similarly, Arias-de la Torre et al. [21] reported lower but non-significant mortality rates for UKA compared to TKA at 5 years in the Catalan registry. Moore et al. [19] identified a 6-fold increase in 30-day mortality risk among octogenarians undergoing UKA compared to younger patients, though absolute mortality remained low. This finding emphasized the elevated baseline mortality risk in octogenarians regardless of procedure type, reinforcing our observation that age—not implant choice—is the primary driver of mortality in this population.

While our study focused on mortality, functional outcomes and quality of life are equally important considerations when selecting between UKA and TKA in elderly patients [22–25]. Goh et al. [9] demonstrated that octogenarians undergoing medial UKA achieved similar improvements in knee-specific function compared to younger patients, though physical well-being scores remained lower at 2 years [9]. Importantly, they found no increase in complications, reoperations, or readmissions within the first postoperative year, with most octogenarians discharged home following outpatient UKA. They also reported a 5-year survivorship of 93% in octogenarians undergoing UKA, demonstrating that when appropriate selection criteria are applied in contemporary practice, revision risk remains acceptably low even in this age group [9].

Furthermore, UKA offers several theoretical advantages particularly important for elderly, medically fragile patients: shorter operative time, reduced blood loss, bone and ligament preservation, faster postoperative mobilization, and superior restoration of native kinematics [4, 9–29]. Moore et al. [19] observed significantly shorter operative times and reduced use of general anesthesia in octogenarian UKA patients, factors potentially contributing to lower cardiac complication rates despite higher baseline comorbidity burden.

The traditional debate surrounding UKA versus TKA centers on differential revision rates. Multiple large registry studies consistently demonstrate higher relative revision rates for UKA compared to TKA, although the absolute revision risk of contemporary, well-selected constructs remains low [20, 21]. Hunt et al. [20] reported 10-year cumulative revision rates of 11.4% for UKA versus 4.4% for TKA, estimating 70 excess revisions per 1000 UKA cases. Importantly, it has been showed that this disparity is volume-dependent: high-volume UKA centers achieved revision rates comparable to TKA, while low-volume centers demonstrated substantially elevated UKA revision risk [21, 30–32].

In the NJR 22nd Annual Report (2025) [33], the best-performing fixed-bearing UKA construct showed a 10-year cumulative revision rate of 4.81% (Physica ZUK medial, cemented; *n* = 32,539), with several widely used constructs achieving comparable rates, although revision risk varied across designs. When cumulative revision was stratified by age, the ≥ 80-year cohort demonstrated the lowest revision rate of any age group, plateauing at approximately 3.6% in men and 7% in women over long-term follow-up. These age-specific data supported our results. In octogenarians, the absolute risk of implant revision is low and is substantially exceeded by competing mortality risk, such that long-term implant durability is unlikely to be the limiting factor in this population.

However, this revision-centric paradigm becomes increasingly questionable when applied to octogenarians. In our cohort, the 35.3% mortality rate at 5–7 years far exceeds the 10-year revision rates reported even in registry studies [13, 14, 20]. This fundamental observation—that octogenarians are far more likely to die than to experience implant failure—reframes the risk-benefit calculus. For every 100 octogenarian UKA patients followed for a median of 6 years, we would expect approximately 35 deaths compared to 7–11 revisions based on registry projections. Put differently, patient mortality is 3 to 5 times more likely than implant revision in this demographic.

Our findings suggest a paradigm shift in how implant selection should be approached in octogenarians. The traditional emphasis on long-term implant survivorship, while appropriate for younger patients with decades of life expectancy, becomes progressively less relevant as mortality risk eclipses revision risk. In octogenarians, where 5- to 6-year mortality approaches 30–60% even in medically optimized cohorts, the likelihood of outliving one’s prosthesis diminishes substantially. When competing mortality risk substantially outweighs revision risk, the clinical imperative shifts from maximizing theoretical implant longevity to optimizing short-term recovery, minimizing surgical trauma, and maximizing functional independence during remaining lifespan. UKA offers procedural advantages particularly relevant in this population: shorter hospital stay, reduced blood loss and transfusion requirements, and lower infection rates [4, 9, 28, 34]. When mortality risk substantially exceeds revision risk, optimizing the immediate perioperative period becomes the rational priority. Whether these clinical advantages translate into healthcare system cost-effectiveness warrants formal economic evaluation.

As the octogenarian population continues to expand at an unprecedented rate, these findings have important implications for the growing volume of knee arthroplasties performed in this demographic. The rationale for choosing the less invasive UKA in elderly patients rests on the principle that if medium-to-long-term outcomes are equivalent between procedures, octogenarian patients may complete their natural lifespan with a well-functioning UKA given their limited life expectancy [34]. This “one-shot solution” philosophy acknowledges that while failed UKA requiring conversion would be disadvantageous, registry evidence demonstrates equivalent UKA survival to TKA when performed by experienced, high-volume surgeons [34].

The convergence of evidence—equivalent mortality between procedures, acceptable revision rates in experienced hands, superior functional recovery characteristics, and reduced surgical invasiveness—supports judicious UKA use in this demographic when clinical indications are met. Importantly, when patient survival is equivalent between procedures and mortality risk outweighs revision risk, surgeons can focus on patient-specific indications (disease pattern, ligamentous stability, deformity) rather than theoretical durability concerns. This paradigm shift—from implant survivorship to patient survivorship—may improve outcomes by matching each patient to the most appropriate, least invasive procedure. If UKA’s procedural advantages reduce perioperative complications and hospital resource utilization, cumulative healthcare costs may favor UKA in appropriately selected octogenarians, though this requires formal cost-effectiveness analysis.

Several limitations warrant acknowledgment. The relatively small sample size) compared to large registry studies may have limited statistical power to detect clinically meaningful differences in mortality between implant types. Baseline imbalance in age between groups, though modest in absolute terms and very marginally significant, introduced potential confounding by indication despite statistical adjustment. The absence of exact death dates precluded conventional time-to-event survival analysis, necessitating binary logistic regression with adjustment for follow-up duration. The retrospective design introduced potential unmeasured confounding. Finally, generalizability may be limited to high-volume centres with experienced surgeons performing UKA in carefully selected patients.

Future prospective studies incorporating comprehensive comorbidity assessment, functional outcome measures, and patient-reported outcomes would provide a more holistic comparison of UKA versus TKA in octogenarians. Competing risk analyses formally incorporating both mortality and revision data would quantify the trade-off between patient survival and implant durability in octogenarians. The identification of subgroups most likely to benefit from UKA could refine patient selection algorithms and improve shared decision-making. Cost-effectiveness analyses comparing UKA versus TKA in octogenarians are needed, incorporating operative time, hospital stay, transfusion requirements, complication rates, and revision burden.

## Conclusion

In octogenarian patients, patient mortality substantially exceeds implant revision risk at mid-term follow-up. Surgeons should prioritize patient-specific clinical indications rather than theoretical long-term implant survivorship concerns when selecting between UKA and TKA. When appropriate indications are met and procedures are performed at high-volume centers, UKA represents a viable, less invasive alternative that can optimize short-term outcomes during the patient’s remaining lifespan.

## Data Availability

The datasets used and/or analyzed during the current study are available from the corresponding author on request.
